# Training of female health workers (FHWs) to enhance the advocacy and communication skills on COVID-19 vaccine and routine Immunization in District Peshawar Khyber Pakhtunkhwa (KP) Pakistan in 2023—a report from the field

**DOI:** 10.3389/fgwh.2025.1355781

**Published:** 2025-02-05

**Authors:** Magid Al-Gunaid, Zakir Hussain, Leen Daoud, Khurram Akram

**Affiliations:** ^1^ Public Health Programs, GHD|EMPHNET, Amman, Jordan; ^2^ Country Offices, GHD|EMPHNET, Islamabad, Pakistan

**Keywords:** COVID-19, routine EPI, advocacy, female health workers, vaccine hesitancy, Peshawar Eastern Mediterranean Region

## Abstract

**Introduction:**

In 2021, a comprehensive evaluation was conducted across five countries, including Pakistan, to explore the factors influencing the demand for COVID-19 vaccines among priority groups. The study uncovered significant challenges, including low vaccination rates among females, limited trust in the COVID-19 vaccine, accessibility issues, and a notable gap in dose administration.

**Methods:**

To address these challenges, a targeted pilot intervention was proposed in Peshawar, Pakistan. This intervention aimed to enhance vaccine demand among young women (aged 18–24), including pregnant and lactating women (PLWs), by equipping approximately 300 female health workers (FHWs) with improved advocacy and communication skills. These skills were designed to combat vaccine hesitancy and increase vaccine acceptance among women. Moreover, efforts were made to strengthen social support from community leaders. The pilot initiative encompassed baseline and endline evaluations to assess its impact. The baseline evaluation involved analyzing existing vaccination data, disaggregated by age and gender. Key informant interviews (KIIs) were conducted to capture qualitative insights into the perceptions of vaccines within the target population.

**Findings:**

Data from the Department of Health KP and Expanded Program on Immunization Management Information System (EPIMIS) highlighted improvements in both COVID-19 and routine Expanded Program on Immunization (EPI) vaccinations across 25 union councils (UCs) in district Peshawar. KIIs with stakeholders, including health workers and community influencers, revealed enhanced knowledge and willingness to vaccinate, particularly among PLWs and females aged 18–24. The evaluation also observed increased confidence and reduced vaccine hesitancy due to advocacy sessions. Additionally, training of FHWs significantly improved their knowledge and attitudes towards COVID-19 vaccination and routine EPI, contributing to the overall success of the intervention. Monitoring visits further validated the effective conduct of advocacy sessions by trained health workers, leading to increased vaccination uptake in the community.

**Conclusion:**

The comprehensive approach undertaken in this pilot intervention aimed not only to improve vaccine uptake but also to bolster confidence in the COVID-19 vaccine within the community. The findings and outcomes of this initiative provided valuable insights for future public health strategies, particularly in addressing vaccine hesitancy and increasing vaccine acceptance among priority groups.

## 1 Introduction

The COVID-19 pandemic has been a defining global health crisis, particularly impacting low- and middle-income countries (LMICs) like Pakistan, a country in the Eastern Mediterranean Region (EMR). The pandemic not only presented direct health challenges but also amplified existing disparities in healthcare access, including in immunization efforts against COVID-19 (1). In Pakistan, these disparities were stark, with vaccination campaigns facing obstacles such as infrastructural challenges, socio-economic barriers, and gender-related hurdles, leading to lower immunization rates and an increased risk of vaccine-preventable diseases (2, 3).

In early 2020, the pandemic severely disrupted routine health services, including the Expanded Programme on Immunization (EPI), aggravating the situation in countries like Pakistan (1, 4). An extensive assessment conducted in 2021 in the EMR, including Pakistan, highlighted significant gender disparities in vaccine uptake. This assessment found that only 39% of unvaccinated females in Pakistan intended to receive the COVID-19 vaccine, compared to 56% of unvaccinated males (5). These disparities were attributed to various factors, including limited healthcare access, vaccine hesitancy, and socio-cultural constraints, particularly affecting women's health choices (5).

To address these challenges, ideation workshops were conducted across four provinces of Pakistan, including stakeholder representation from health authorities and Non-governmental Organizations (NGOs). These workshops, informed by the assessment findings, underscored the need for gender-responsive strategies in vaccination programs (6). The discussions highlighted the urgent need to tailor immunization strategies to local contexts, with a focus on overcoming socio-cultural barriers and enhancing access for underserved groups, especially women.

The pilot intervention was designed in response to these insights was implemented in the Peshawar district of Khyber Pakhtunkhwa (KP) province. This intervention focused on building the capacity of Female Health Workers (FHWs) in advocacy and communication skills to combat vaccine hesitancy and increase vaccine uptake, particularly among pregnant and lactating women (PLWs) and women aged 18–24. The FHWs, crucial in connecting communities with healthcare systems, were trained to address the specific challenges faced by women in accessing vaccines (7). This initiative was aligned with the global emphasis on gender mainstreaming in public health responses, particularly in the context of the COVID-19 pandemic (8).

We have also categorized the themes derived from our KIIs to specify the different types of informants involved, such as community leaders, healthcare workers, and women receiving the intervention. This helps provide a clearer picture of the unique perspectives of these stakeholder groups. In addition, representative verbatim quotes from KIIs have been included in relevant sections to provide richer context to the identified themes, ensuring that the unique voices and concerns of different informants are effectively highlighted.

This report presents a comprehensive overview of the methodology, implementation, and outcomes of the intervention in District Peshawar. It aims to provide insights into the effectiveness of gender-focused strategies in enhancing vaccine uptake during the COVID-19 pandemic, contributing to the broader discourse on integrating gender perspectives into global health initiatives, especially in the realm of immunization.

## 2 Methodology

### 2.1 Assessment and pilot intervention design

The foundation of the pilot intervention was laid by a comprehensive assessment carried out in 2020 across five countries, including Pakistan. This assessment, funded by the CDC and executed in partnership with local NGOs, was pivotal in identifying barriers and facilitators to COVID-19 vaccine uptake among the priority groups. Employing a mixed-methods approach, the assessment combined quantitative data from health records and surveys with qualitative insights from KIIs and focus groups and in-depth interviews ensuring a holistic understanding of the challenges faced in the EMR.

The pilot intervention in District Peshawar, KP, Pakistan, emerged from these findings, with a specific focus on young women aged 18–24, including PLWs. Recognizing the key role of FHWs in community health, the intervention comprised several activities:

1. Training of FHWs: Targeting 300 FHWs, the training program aimed to enhance their skills in advocacy and communication regarding COVID-19 and routine immunization. The training included modules on:
  a. Understanding COVID-19 vaccines: Efficacy, safety, and public health importance.
  b. Inter-personal Communication techniques: Addressing concerns, myth-busting, and effective messaging.
  c. Community engagement: Strategies to engage different demographic groups, with a focus on women and PLWs.
2. Community Engagement and Advocacy Sessions: FHWs conducted sessions in communities, focusing on areas with low vaccine uptake. These sessions included:
  a. Group discussions and information sessions.
  b. Home visits to engage with families, husbands and mothers-in-law, especially targeting women 18–24 years old and PLWs.
  c. Collaborations with local leaders and influencers to enhance community-wide awareness and support.
3. Supervisory and Monitoring Activities: Supervisory team meetings were established to monitor and guide FHWs. Activities included:
  a. Regular monitoring field visits to assess the progress of FHWs in the field.
  b. Monthly meetings with FHWs to refine strategies and address challenges.
  c. Data collection on outreach efforts and community responses.

### 2.2 Training program for FHWs

The training program was meticulously designed to empower FHWs with the necessary knowledge and skills. It was structured into several sessions, each focusing on specific aspects:

1. Module on Vaccine Science: Detailed information about COVID-19 vaccines, including their development, efficacy, safety profiles, and the importance of routine EPI.
2. Communication Skills Development: Focused on enhancing interpersonal communication skills, addressing vaccine hesitancy, and fostering trust in healthcare advice.
3. Community Engagement Methods: Training on organizing and conducting effective community sessions, home visits, and engaging with diverse groups within the community.

### 2.3 Data collection methods for baseline and endline evaluations

The evaluation framework was designed to capture both the process and outcomes of the intervention:

1. Baseline Evaluation: Involved analyzing pre-existing vaccination data, disaggregated by age and gender. Additionally, 15 KIIs were conducted, adopting a purposive sampling methodology to capture qualitative insights into vaccine perceptions. The 15 KII participants provided written informed consent to participate in the evaluation.
2. Endline Evaluation: Employed similar metrics as the baseline to evaluate changes in vaccine uptake and confidence levels.
3. Process Evaluation: Focused on quantifying the intervention's reach and effectiveness. Metrics included the number of training sessions conducted, the number of advocacy sessions held, and the number of PLWs reached.
4. Qualitative Assessments: KIIs were repeated at the endline to assess shifts in community perceptions and the impact of FHWs' activities. Written informed consent to participate in the endline evaluation was obtained from the KII participants.

## 3 Findings

### 3.1 Baseline findings

COVID-19 vaccine coverage data in Khyber Pakhtunkhwa (KP) was obtained from Department of Health KP's situation reports. As of June 30th, 2023, the eligible adult population for COVID-19 vaccination was 12,564,421, with 4,870,700 in the 12–17 years age group and 7,693,721 in the 18–29 years age group. In district Peshawar, around 2 million individuals were vaccinated against COVID-19 while 1.8 million received second dose and 0.86 million received booster dose.

**Figure 1:**
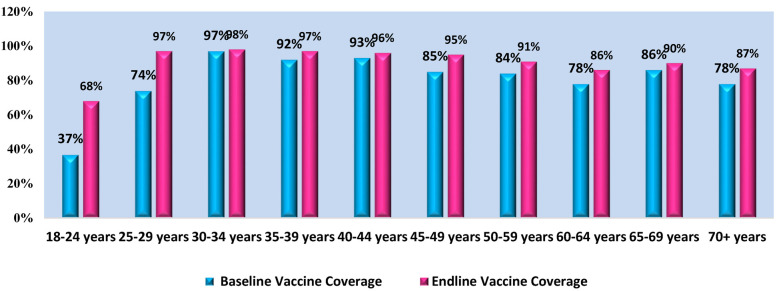
Comparative analysis of COVID 19 vaccination - pre & post intervention.

It is evident that in all age groups, on an average there is 6%–9% increase in the COVID-vaccination post-intervention. Figure 1 illustrates the comparative analysis of COVID-19 vaccination pre and post intervention. However, there is a remarkable increase of 31% and 23% from the baseline vaccination in the adolescent age group of 18–24 and 25–29 years, respectively. This overall increase can attribute to the awareness and advocacy sessions conducted by the FHWs in their respective communities and producing a positive impact and thus an improvement. Table 1 shows the age-wise breakdown of the pre- and post-intervention for COVID-19 vaccination.

**Table 1:** Age-wise breakdown of the pre-and post-intervention COVID 19 vaccinations.

Age group	COVID-19 vaccine coverage (%) Baseline	COVID-19 vaccine coverage (%) Post Intervention
18–24 years	37	68
25–29 years	74	97
30–34 years	97	98
35–39 years	92	97
40–44 years	93	96
45–49 years	85	95
50–59 years	84	91
60–64 years	78	86
65–69 years	86	90
70+ years	78	87

### 3.2 Routine EPI coverage findings

The routine EPI coverage data for the targeted 25 union councils of district Peshawar, KP for the antigens BCG, OPV and Penta III was gathered from the Expanded Program on immunization Management Information System (EPIMIS). The pre-intervention cumulative data for age groups 0–11 months and 12–23 months in last quarter of 2022 (Sep–Dec) was compared with the post-intervention routine EPI coverage of the same cohort, from Feb-May 2023. The results are as follows:

The comparison of the consolidated UC wise data of age group 0–11 months age during the pre and post intervention periods, Sep–Nov 2022 and Feb–May 2023 reveals that there is an increase in the BCG vaccination from 75% to 88%, and 71% to 100% in UC Adezai, Deh Bahadar Kallay, respectively. Likewise, the OPV 0 dose was also increased in UC Deh Bahadar Kallay and UC Mattani from 71% to 98%, 65% to 73%, respectively. The vaccination coverage was also increased in UC Yakatoot 2, from 77% to 86% for BCG, 82% to 86% for OPV 0% and 71% to 100% for OPV II. Thus, an overall improvement was noted in routine EPI vaccination coverage in post-intervention reporting period.

The comparison of the consolidated UC wise routine EPI coverage data of age groups 12–23 months age during the pre and post intervention periods Sep–Nov 2022 and Feb–May 2023 reveals that there was an increase in the OPV I vaccination from 0% to 11%, 12% and 14% in UCs Deh Bahadar Kallay, Kaniza and Nahaqi, respectively. Likewise, the OPV II dose intake was also increased in UCs Gulbela, Kaniza and Nahaqi from 0% to 4%, 9% and 18%, respectively. The Penta III vaccination has also increased in UCs Yakatoot 2 and Nahaqi, from 0% to 4% and 11%, respectively. Thus, an overall improvement was noted in routine EPI vaccination coverage in post-intervention reporting period. Table 2 shows the comparative analysis of the baseline and endline COVID-19 coverage among different age groups. Tables 3–5 show the UC-wize EPI coverage both pre- and post intervention.

**Table 2:** Comparative analysis of baseline and endline COVID 19 vaccination among different age groups.

S#	Union council	BCG%	OPV%				PENTA III%
			0	I	II	III	
1	Ade Zai	75	117	99	81	86	98
2	Badaber Huri Zai	128	128	122	105	101	101
3	Badaber Maryam Zai	138	115	129	118	89	123
4	Budni	105	105	127	102	105	104
5	Deh Bahader Kally	71	71	92	67	88	85
6	Gulbela	109	108	125	118	126	122
7	Hazarkhani 2	107	87	113	105	107	107
8	Kaniza	128	106	131	107	143	143
9	Khazana	160	133	173	142	127	125
10	Maryam Zai	116	93	122	109	118	118
11	Mashogagar	120	94	115	90	112	112
12	Mathra	109	95	130	112	116	116
13	Mattani	103	69	101	91	83	100
14	Nahaqi	110	103	122	113	120	133
15	Pajagai/Faqir Kalli	119	118	148	146	147	147
16	Pakha Ghulam	99	99	99	101	92	92
17	Palosi	129	113	168	140	153	153
18	Panam Dehri	103	99	139	100	137	137
19	Sheikh Junaid Abad	85	85	102	106	103	95
20	Tehkal Payan - 1	126	126	114	99	115	115
21	Urmar Bala	105	89	92	85	90	90
22	Urmar Mina	129	108	101	80	86	86
23	Urmar Payan	117	117	110	109	104	104
24	Wadpaga	114	110	121	119	126	116
25	Yakatoot 2	77	82	120	71	96	123

**Table 3:** UC wise post-intervention routine EPI coverage among the age group (0–11 months) Feb–May 2023.

	Union council	BCG%	OPV%				PENTA III%
			0	I	II	III	
1	Ade Zai	88	85	78	69	74	76
2	Badaber Huri Zai	96	98	93	89	85	85
3	Badaber Maryam Zai	131	134	108	101	100	100
4	Budni	87	87	101	88	91	90
5	Deh Bahader Kally	100	98	99	80	90	90
6	Gulbela	88	77	100	97	95	95
7	Hazarkhani 2	130	130	119	108	113	109
8	Kaniza	86	78	97	91	100	101
9	Khazana	135	124	106	101	101	101
10	Maryam Zai	104	91	82	76	78	78
11	Mashogagar	95	73	82	62	68	90
12	Mathra	111	94	124	112	97	101
13	Mattani	85	73	99	94	94	93
14	Nahaqi	106	101	102	100	101	101
15	Pajagai/Faqir Kalli	100	94	107	109	91	91
16	Pakha Ghulam	89	88	104	98	94	100
17	Palosi	157	115	115	109	102	98
18	Panam Dehri	99	99	105	88	101	101
19	Sheikh Junaid Abad	76	76	109	99	96	96
20	Tehkal Payan - 1	112	112	99	92	95	95
21	Urmar Bala	90	82	91	90	85	85
22	Urmar Mina	89	88	88	84	85	86
23	Urmar Payan	100	100	109	101	101	101
24	Wadpaga	77	75	105	103	100	99
25	Yakatoot 2	86	86	117	100	105	106

**Table 4:** UC wise pre-intervention routine EPI coverage aged (12–23 months) Sep–Nov 2022.

	Union council	BCG%	OPV%				PENTA III%
			0	I	II	III	
1	Ade Zai	0	0	0	0	0	0
2	Badaber Huri Zai	0	0	0	0	0	0
3	Badaber Maryam Zai	0	0	0	0	0	0
4	Budni	0	0	0	0	0	0
5	Deh Bahader Kally	0	0	0	0	0	0
6	Gulbela	0	0	0	0	0	0
7	Hazarkhani 2	0	0	0	0	0	0
8	Kaniza	0	0	0	0	0	0
9	Khazana	0	0	0	0	0	0
10	Maryam Zai	0	0	0	0	0	0
11	Mashogagar	0	0	0	0	0	0
12	Mathra	0	0	0	0	0	0
13	Mattani	0	0	0	0	0	0
14	Nahaqi	0	0	0	0	0	0
15	Pajagai/Faqir Kalli	0	0	0	0	0	0
16	Pakha Ghulam	0	0	0	0	0	0
17	Palosi	0	0	0	0	0	0
18	Panam Dehri	0	0	0	0	0	0
19	Sheikh Junaid Abad	0	0	0	0	0	0
20	Tehkal Payan - 1	0	0	0	0	0	0
21	Urmar Bala	0	0	0	0	0	0
22	Urmar Mina	0	0	0	0	0	0
23	Urmar Payan	0	0	0	0	0	0
24	Wadpaga	0	0	0	0	0	0
25	Yakatoot 2	0	0	11	0	0	0

**Table 5:** UC wise post-intervention routine EPI coverage aged (12–23 months) Feb–May 2023.

	Union council	BCG%	OPV%				PENTA III%
			0	I	II	III	
1	Ade Zai	0	0	1	0	0	0
2	Badaber Huri Zai	0	0	3	3	4	4
3	Badaber Maryam Zai	0	0	2	4	2	2
4	Budni	0	0	0	1	0	0
5	Deh Bahader Kally	0	0	11	4	9	9
6	Gulbela	0	0	4	4	5	5
7	Hazarkhani 2	0	0	0	0	0	0
8	Kaniza	0	0	12	9	8	8
9	Khazana	0	0	1	1	1	1
10	Maryam Zai	0	0	2	2	2	2
11	Mashogagar	0	0	0	0	0	0
12	Mathra	0	0	3	1	4	4
13	Mattani	0	0	0	0	0	0
14	Nahaqi	0	0	14	18	11	11
15	Pajagai/Faqir Kalli	0	0	5	4	5	6
16	Pakha Ghulam	0	0	4	1	1	1
17	Palosi	0	0	0	0	0	0
18	Panam Dehri	0	0	1	0	0	0
19	Sheikh Junaid Abad	0	0	0	0	0	0
20	Tehkal Payan - 1	0	0	1	1	2	2
21	Urmar Bala	0	0	0	0	0	0
22	Urmar Mina	0	0	0	0	0	0
23	Urmar Payan	0	0	0	0	0	0
24	Wadpaga	0	0	0	0	0	0
25	Yakatoot 2	0	0	1	1	4	4

### 3.3 Key informant interviews (KIIs) findings

#### 3.3.1 Participant's demography

Purposive sampling methodology was adopted to conduct KIIs with 20 key stakeholders during post-intervention period from 30th May to 8th June 2023 in district Peshawar, KP. The participants were comprised of the following with the mean age of 40 ± 6.34 years.

1. Community influencers; the community leaders, mothers-in-law, husbands, and PLWs – 8 KIIS
2. Health workers – 6 KIIs
3. Vaccinators – 4 KIIs
4. District EPI focal person – 1 KII
5. LHW Program Coordinator – 1 KII

The following themes were assessed through KIIs with key stakeholders:

1. Level of knowledge about COVID 19 vaccine
2. Intention and willingness to receive COVID vaccine.
3. COVID-19 vaccine confidence and hesitancy among PLWs and females aged 18–24.
4. COVID-19 vaccination intention among PLWs and females aged 18–24
5. Routine EPI Coverage and awareness among community influencers, mothers-in-law, and husbands
6. Status of referrals by FHWs for PLWs and females aged 18–24.

#### 3.3.2 Knowledge about COVID 19 vaccine

The study observed that age, education, domicile, and employment position had a significant impact on the knowledge and perception score.

#### 3.3.3 Intention/willingness to get COVID-19 vaccine

It was observed that individuals with higher literacy rates, regardless of gender, displayed a greater willingness to receive vaccinations compared to those who were illiterate or had lower levels of education. The primary apprehension and hesitancy revolved around breastfeeding, misconceptions, and rumors regarding the COVID vaccine, as well as the financial implications of vaccination.

#### 3.3.4 COVID-19 vaccine confidence and hesitancy among PLWs and females aged 18–24

The KIIs revealed that the vaccine hesitancy was effectively addressed by the advocacy sessions conducted during the intervention. The key informants were able to relate to many concerns regarding vaccine safety, efficacy, and mistrust in the healthcare delivery system. However, the monthly advocacy sessions and community engagement were the successful activities which achieved its targets through education and communication. It was further validated by the EPIMIS KP data of EPID week 22–24, in which zero COVID cases were reported in district Peshawar. This can be attributed to the enhanced uptake of the COVID vaccine due to the advocacy sessions conducted during the intervention.

#### 3.3.5 COVID-19 vaccination intention among PLWs and females aged 18–24

The FHWs reported that the PLWs and females aged 18–24 in the respective catchment population have shown an improvement in confidence in receiving COVID-19 vaccines. Their perception has improved after attending the advocacy sessions and now consider the COVID vaccination as an opportunity to protect themselves and their infants from severe illness or complications associated with COVID-19.

#### 3.3.6 Routine EPI coverage and awareness among community influencers, mothers-in-law, and husbands

The awareness regarding routine EPI has improved the decision-making process for vaccination uptake by enhancing knowledge, building trust, addressing misconceptions, improving accessibility, and engaging communities. The advocacy sessions involving the key stakeholders, such as local leaders, healthcare providers, and community organizations, amplified the impact of routine EPI. It has improved the understanding, addressed concerns, and increased vaccination coverage which was also validated during the desk review of EPIMIS data.

#### 3.3.7 Status of referrals by FHWs for PLWs and females aged 18–24

The analysis revealed that intervention has strengthened the capacity of the LHWs to identify various health issues and immunization concerns within their communities. The community influencers reported that the intervention has also guided the Lady Health Workers (LHWs) to maintain a regular follow-up with the individuals they have referred to. This was validated by reviewing their monitoring tools. This ensures continuity of care by monitoring the immunization status of children, reinforce adherence to ante-natal, natal and post-natal plans of pregnant and lactating mothers. This follow-up helps improve health outcomes and encourages individuals to continue seeking appropriate healthcare services.

### 3.4 Knowledge assessment of FHWs

The baseline assessment data indicated that a total of 313 female health staff, comprising of LHWs, lady health visitors (LHVs), community midwives (CMWs) and lady health supervisors (LHS) participated in the training and completed the pre and post-test. The mean age was 39 ± 6.93 (range: 24–61 years). All (*n* = 313) of the participants were females. Majority 82% of the participants had secondary school and higher secondary school level education (*n* = 246) while 5% (*n* = 15) of the participants had graduation level education and 3% (*n* = 9) of the participants had master's level education. 78% participants (*n* = 244) were LHWs, 7% participants (23) were community midwives (CMWs), 6% participants (19) were LHVs, and 8% participants were lady health supervisors (LHSs). Pashto is the native language of 81% of the participants (*n* = 250) while 10% participants (*n* = 30) speak Hindko language. Amongst the 313 participants, a sample of 20 participants, comprised of 5 LHS, 5 LHW, 5 LHVs and 5 CMWs were selected and the questionnaire used during pre-post teat was administered to determine effectiveness of the training in improving FHW's knowledge and attitudes regarding COVID-19 vaccination and routine immunization, and their understanding of the COVID-19 disease, transmission, high-risk groups, vaccine benefits, safety, side effects, as well as topics related to vaccine hesitancy and advocacy.

**Figure 2:**
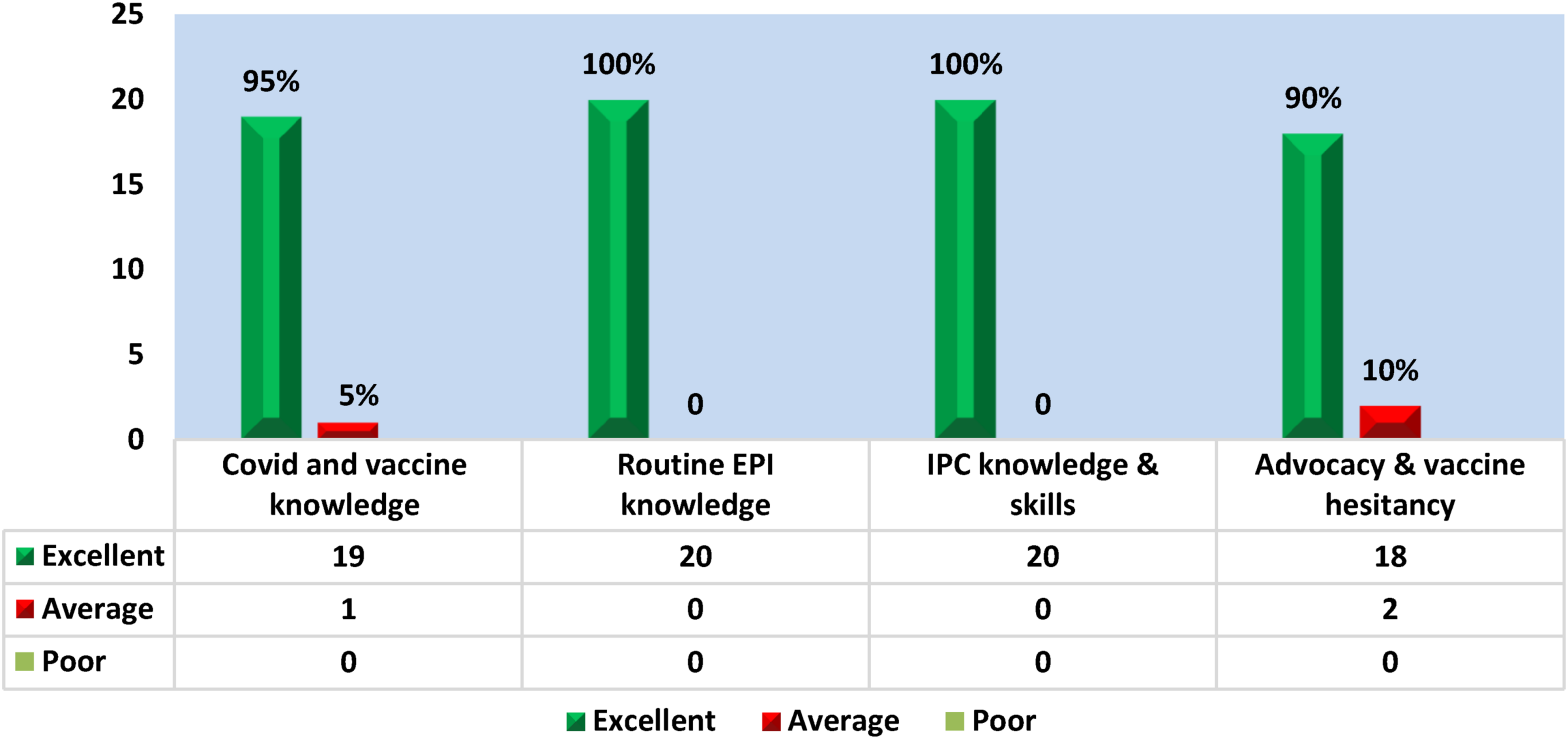
Endline assessment of FHW knowledge.

The results revealed that amongst the sample of 20 participants, the questions regarding routine EPI and IPC knowledge and skills were answered correctly by all the respondent (*n* = 20). Whereas 95% (*n* = 19) were able to respond accurately about the COVID-19 and vaccine knowledge. The advocacy and vaccine hesitancy were responded correctly by 90% (*n* = 18) as illustrated in Figure 2.

This highlights that the capacity building of the FHWs as a part of the intervention had a positive impact on the knowledge, attitudes, and practices of the FHWs regarding COVID 19 and routine EPI.

### 3.5 Monitoring and supervisory visits

Monitoring of the advocacy sessions by the trained FHWs with the target groups was carried out fortnightly by the technical teams. Issues observed during these monitoring visits were resolved with the support of district health office Peshawar. During these monitoring visits it was observed that the trained FHWs conducted advocacy sessions in a structured manner and a good number of community members attended these advocacy sessions. Through these advocacy sessions FHWs were able to identify unvaccinated PLWs, women aged 18–24 years and children less than 2 years of age. Some of the sessions were arranged in schools where these FHWs had session with the teachers and students with the objective to mitigate negative information related to the vaccination.

## 4 Discussion

1. Leveraging Behavioral Change for Enhanced Vaccine Uptake:
  The intervention in Peshawar, KP, Pakistan, exemplifies the profound impact of behavioral change strategies in public health initiatives, especially in the context of vaccine uptake. By focusing on training FHWs and empowering them as community advocates, the intervention successfully shifted vaccine perceptions among women, a critical demographic in the region. The nuanced understanding and respect for local cultural norms exhibited by FHWs, coupled with their newfound advocacy skills, facilitated a deeper penetration of accurate health messages within the community. Additionally, this approach helped build trust within the community, reducing resistance and increasing acceptance of vaccines. This approach highlights the importance of not just disseminating information, but ensuring it resonates with the target audience's cultural and social realities.
2. Addressing Gender Disparities in Health Access:
  In LMICs like Pakistan, gender disparities often manifest starkly in healthcare access and utilization. The intervention's focus on women, particularly young and PLWs, directly addressed these disparities, bridging a crucial gap in the COVID-19 response. By enabling FHWs to specifically target women through home visits, community sessions, and collaborative efforts with local influencers, the initiative acknowledged and effectively responded to the unique barriers faced by women in accessing healthcare. This gender-sensitive approach not only improved vaccine uptake but also fostered a sense of empowerment among women, contributing to broader goals of gender equity in healthcare. Future interventions should consider expanding similar approaches to address other forms of health access disparities, particularly those affected by socio-economic constraints and decision-making power within households.
3. Combatting Vaccine Hesitancy and Misinformation:
  A critical challenge in the COVID-19 vaccination campaign has been the pervasive spread of misinformation and hesitancy. The intervention tackled this challenge head-on through a two-pronged approach: First, by providing FHWs with accurate, up-to-date information on COVID-19 vaccines, equipping them to be reliable sources of information. Second, by training them in communication strategies to effectively counteract rumors and misinformation. This approach emphasized the importance of not only correcting false information but also building trust and understanding within the community. The success of these strategies was evident in the improved vaccine confidence observed at the end of the intervention, demonstrating the power of well-informed, community-level advocacy in changing health behaviors. Further efforts should include integrating digital tools to counter misinformation, enabling FHWs to reach broader audiences and provide real-time responses to emerging myths and concerns.
4. Context-Specific Strategies for LMIC Settings:
  The intervention's success was largely due to its context-specific design, which was carefully tailored to meet the unique needs and challenges of the target population in Peshawar. This contextualization is crucial in LMIC settings, where diverse socio-cultural landscapes and resource constraints require innovative, localized solutions. The intervention's model, focusing on community engagement and empowerment of local health workers, provides a replicable blueprint for similar settings. Future efforts should incorporate adaptability assessments to ensure that core components of the intervention can be effectively modified to suit new contexts, accounting for cultural, economic, and logistical differences. It underscores the importance of going beyond one-size-fits-all approaches and developing strategies that are deeply rooted in the local context.
5. Expanding the Scope of Public Health Interventions:
  The findings from Peshawar extend beyond the immediate context of COVID-19 vaccination. They offer insights into how similar strategies can be employed for other public health challenges, particularly those where behavioral change is a key component. The role of FHWs as change agents can be leveraged in various areas, from routine immunization to maternal and child health, underscoring the value of investing in local health workforces as a long-term public health strategy. Additionally, targeted training on new and emerging health priorities, such as non-communicable diseases and mental health, could further enhance the scope and impact of FHWs.
6. Future Directions and Recommendations:
  Based on the intervention's outcomes, future initiatives should consider the following:
  a. Continued Training and Support for FHWs: Sustained investment in training and supporting FHWs is essential for the continued success of such interventions. In particular, refresher courses and continuous learning opportunities can help FHWs stay updated on best practices, ensuring they remain effective advocates in their communities.
  b. Expansion to Other Regions and Health Areas: Replicating this model in other regions and for different health challenges could yield significant public health benefits. Future interventions should consider how best to adapt the methods to fit local contexts without losing the core elements that led to success in Peshawar. Specifically, these interventions should incorporate local stakeholder engagement early in the planning stages to ensure cultural relevance and buy-in.
  c. Long-Term Monitoring and Evaluation: Ongoing evaluation is crucial to assess the long-term impact and sustainability of behavioral changes induced by such interventions. Regular monitoring will help ensure that positive changes are sustained and can also highlight areas where further support is needed. Moreover, tracking metrics should include not just vaccine uptake but also community trust in healthcare services, which is key to achieving sustained health improvements.
  d. Incorporation of Digital Technologies: Exploring digital platforms for training and community engagement can expand the reach and effectiveness of health interventions. Mobile applications and digital tools can facilitate real-time tracking, feedback, and communication, making health interventions more responsive and adaptive. These tools could also be leveraged for ongoing education for FHWs and for gathering community feedback to improve intervention strategies.
  e. Policy Integration: Integrating learnings from this intervention into broader health policies can help institutionalize effective strategies for behavior change and gender mainstreaming in public health. Policymakers should consider incorporating FHW empowerment models into national immunization programs to ensure widespread adoption of successful methods. Such integration can also include establishing formal recognition and incentives for FHWs, which would motivate and sustain their engagement in public health initiatives.

## 5 Limitations

This study, while providing valuable insights, has several limitations that must be acknowledged. Firstly, the duration of the intervention was relatively short, limiting our ability to assess long-term sustainability of the behavioral changes observed. Future studies should incorporate longer follow-up periods to better evaluate the persistence of behavioral changes over time. Secondly, the study focused primarily on one district in Pakistan, and the findings may not be generalizable to other regions with different socio-cultural dynamics. To improve generalizability, future interventions should be tested in a variety of socio-cultural contexts, including urban and rural settings across different provinces. Thirdly, the reliance on self-reported data in surveys and interviews could introduce response biases. In future work, incorporating objective measures, such as health records or observational data, could help mitigate the potential biases associated with self-reporting. Finally, the COVID-19 pandemic's evolving nature and varying public health measures over time may have influenced the study's outcomes and their interpretation. To address this, future research should include control groups and account for changes in public health guidelines to better isolate the effects of the intervention.

Understanding these limitations is crucial for interpreting the study's findings and for the design of future interventions. Future research should aim to address these limitations by incorporating longer follow-up periods, expanding the geographic scope of interventions, and employing more robust data collection methods.

## 6 Conclusion

The intervention conducted in District Peshawar, KP, Pakistan, provides a compelling case study in the efficacy of community-centered, gender-sensitive approaches in public health, especially in the context of a global pandemic. By focusing on the training and empowerment of FHWs, the intervention achieved significant strides in improving COVID-19 vaccine uptake and confidence among women, particularly among young, pregnant, and lactating women. This success highlights the critical role of FHWs as catalysts for change, bridging the gap between healthcare systems and communities.

Key to the intervention's effectiveness was its nuanced understanding of the local context, incorporating culturally and socially relevant strategies to address vaccine hesitancy and misinformation. This culturally tailored approach was instrumental in building trust between healthcare providers and community members, reducing vaccine hesitancy, and ensuring that health messages were delivered in an accessible and resonant manner. This approach underscores the importance of contextualizing health interventions to align with the specific needs and dynamics of the target population. The positive behavioral changes observed, particularly in vaccine perceptions and uptake, demonstrate the potential of such tailored interventions in eliciting meaningful and sustainable health outcomes.

Moreover, the intervention's focus on gender dynamics in health access and decision-making provided valuable insights into how gender mainstreaming can be effectively integrated into immunization programs. By addressing the unique barriers faced by women in accessing healthcare, the initiative not only improved vaccine coverage but also contributed to broader goals of gender equity in health. Future health programs should build on this success by further exploring the intersectionality of gender, socio-economic status, and healthcare access to ensure more inclusive public health strategies.

The lessons learned from this intervention are particularly relevant for LMICs, where resource constraints and diverse cultural landscapes often pose unique challenges to public health initiatives. The success in Peshawar serves as an encouraging model for other regions facing similar challenges. It highlights the potential of leveraging local resources, such as FHWs, and the importance of community engagement in driving public health improvements. The replicability of this model in other LMICs with similar socio-cultural and economic challenges indicates its potential to be scaled up effectively, provided that adaptation to local contexts is prioritized.

Looking forward, the insights gained from this intervention can inform future public health strategies, both within and beyond the realm of immunization. The approach of empowering local health workers, tailoring strategies to specific community needs, and focusing on gender-sensitive interventions has the potential to significantly impact various areas of public health.

The intervention in Peshawar stands as a testament to the power of community-based, gender-focused public health strategies in LMICs. It also emphasizes the importance of policy integration, where successful community-level interventions are scaled through inclusion in national health strategies, thus ensuring sustainability. It offers a scalable and replicable model for addressing health challenges in similar contexts, providing a pathway for more resilient and inclusive health systems in the face of ongoing and future public health emergencies.

## Data Availability

The original contributions presented in the study are included in the article/Supplementary Material, further inquiries can be directed to the corresponding author.

## References

[1] Din M Asghar M Ali M. Delays in polio vaccination programs due to COVID-19 in Pakistan: a major threat to Pakistan’s long war against polio virus. Public Health. (2020) 189:1–2. 10.1016/j.puhe.2020.09.004 33065398 PMC7552970

[2] Owais A Khowaja AR Ali SA Zaidi AK. Pakistan’s expanded programme on immunization: an overview in the context of polio eradication and strategies for improving coverage. Vaccine. (2013) 31(33):3313–9. 10.1016/j.vaccine.2013.05.015 23707167

[3] Khowaja AR Zaman U Feroze A Rizvi A Zaidi AK. Routine EPI coverage: subdistrict inequalities and reasons for immunization failure in a rural setting in Pakistan. Asia Pac J Public Health. (2015) 27(2):NP1050-NP1059. 10.1177/1010539511430850 22186395

[4] Mbaeyi C Baig S Khan Z Young H Kader M Jorba J Progress toward poliomyelitis eradication - Pakistan, January 2020–July 2021. MMWR Morb Mortal Wkly Rep. (2021) 70(39):1359–64. 10.15585/mmwr.mm7039a1 34591827 PMC8486386

[5] Gallup Pakistan. EMR Demand Assessment of COVID-19 Vaccine: Insights from Pakistan. (2021). (Unpublished report submitted to CDC Headquarters on 12 October 2021).

[6] Gallup Pakistan. Comprehensive Report on Ideation Workshops. (2022). (Unpublished reportsubmitted to CDC Headquarters on 11 April 2022).

[7] Hafeez A Mohamud K Shiekh M Shah S Jooma R. Lady health workers programme in Pakistan: challenges, achievements and the way forward. J Pak Med Assoc. (2011) 61:210–5. 21465929

[8] Asi YM Bebasari P Hardy E Lokot M Meagher K Ogbe E Assessing gender responsiveness of COVID-19 response plans for populations in conflict-affected humanitarian emergencies. Confl Health. (2022) 16:4. 10.1186/s13031-022-00435-3 35164797 PMC8842977

